# Fermentation of mixed substrates by *Clostridium pasteurianum* and its physiological, metabolic and proteomic characterizations

**DOI:** 10.1186/s12934-016-0497-4

**Published:** 2016-06-21

**Authors:** Wael Sabra, Wei Wang, Sruthi Surandram, Christin Groeger, An-Ping Zeng

**Affiliations:** Institute of Bioprocess and Biosystems Engineering, Hamburg University of Technology, Denickestrasse 15, 21071 Hamburg, Germany

**Keywords:** Butanol, 1,3-propanediol, Pyruvate decarboxylation, Oxaloacetate, Mixed substrate fermentation

## Abstract

**Background:**

*Clostridium pasteurianum* is becoming increasingly attractive for the production of chemicals and fuels such as n-butanol and 1,3-propanediol. Previously we have shown that dual substrate fermentation using glucose and glycerol enhanced the cell growth and butanol production significantly. Although *C. pasteurianum* can grow efficiently with either glucose or glycerol alone, under certain conditions, glucose limitation in the mixed substrate fermentation leads to growth cessation. To understand this phenomenon and for process optimization, fermentation experiments were performed in the presence of excess glycerol but with varied initial concentrations of glucose which were followed by physiological, metabolic and proteomic analyses.

**Results:**

Physiological characterization showed that the observed cease of growth is not due to the toxicity of n-butanol. Furthermore, the growth can be resumed by addition of glucose or the intermediate oxaloacetate. Proteomic analysis shed more light on the system-level regulation of many proteins directly or indirectly associated with this phenomenon. Surprisingly, it is found that the specific growth rate of *C. pasteurianum* in the different growth phases (e.g. before and after glucose limitation) correlated well with the expression level of the ATP dependent pyruvate carboxylase and with the expression level of biotin synthase which provides the cofactor biotin for the formation of oxaloacetate from pyruvate. Bioenergetic analysis based on the formation rates of metabolites further show that ATP supply is not a limiting factor for the pyruvate carboxylation to oxaloacetate.

**Conclusions:**

The results of physiological and proteomic analyses clearly show that the anaplerotic synthesis of oxaloacetate plays a key role in determining the growth behaviour of *C. pasteurianum* in fermentations with mixed substrates of glucose and glycerol. This study provides interesting targets for metabolic engineering of this emerging industrial microorganism.

**Electronic supplementary material:**

The online version of this article (doi:10.1186/s12934-016-0497-4) contains supplementary material, which is available to authorized users.

## Background

Biological production of chemicals and fuel from renewable resources is an attractive approach to a sustainable future industry. In particular, n-butanol has received increased attention as a potential fuel substitute and an important chemical feedstock. Previously, biofuels have been predominantly produced from crop biomass, resulting in competition with limited food resources and land. Therefore, bacterial fermentation of non-food biomass has been considered a possible answer to this problem [1]. Recent interest in the fermentative route of n-butanol production has led to a large number of studies on the metabolism and genetics of solventogenic *clostridia,* and on the improvement of fermentation and product recovery technologies [1–6]. Currently, there are still three major hurdles for fermentative n-butanol production to compete with the petroleum-based one [7, 8]. These include (a) high cost of substrates, (b) low final product concentrations due to limited bacterial tolerance and therefore, and (c) high product recovery costs. Significant energy savings can be achieved if the concentration of n-butanol in the fermentation broth is increased.

*Clostridium pasteurianum* can produce n-butanol and 1,3-propanediol (1,3-PDO) with completely different patterns from the well-studied *C. acetobutylicum* in the classic aceton-butanol-ethanol (ABE) process [9, 10]. In *C. acetobutylicum, t*he metabolic pathway of ABE fermentation comprises two characteristic phases: acidogenesis and solventogenesis, whereas in *C. pasteurianum* DSMZ 525, n-butanol together with 1,3-PDO is produced in the culture medium from the beginning, and only one phase is detected when grown on glycerol as sole carbon source [10]. Acids (and to a lesser extent n-butanol) are the major products produced if glucose is used as the main carbon source. Mixed-substrate fermentation using glucose and glycerol was shown to be superior for n-butanol production by *C. pasteurianum* [10, 11]. However, limitation of either substrate led to decreased n-butanol formation significantly [10]. The growth pattern was also affected by the nature of substrate used. The highest biomass concentrations were found in experiments with higher glucose concentration (as mono substrate or in blend), followed by with glycerol as mono substrate. Moreover, in mixed substrate fermentation, despite the presence of excess glycerol, limitation of glucose stopped cell growth and limited n-butanol production significantly [10]. The mechanisms behind such phenomenon are not well understood.

In this study we performed mixed substrate fermentations at different initial glucose concentrations. In addition to physiological characterization of cell growth and product formation, comparative proteomic analysis of cultures from different growth phases was performed. Proteomic analysis indicated the importance of the anaplerotic synthesis of oxaloacetate. The ATP and biotin dependent pyruvate carboxylase enzyme is down regulated in the glucose limited phase, and hence may explain the observed growth limitation. Biotin synthesis together with several important enzymes needed for growth was also down regulated in the glucose limited phase. These results provided interesting target for optimization of the growth of *C. pasteurianum* on mixed substrates of glucose and glycerol.

## Results and discussion

### Growth patterns of *C. pasteurianum* in mixed substrate fermentation

**Fig. 1:**
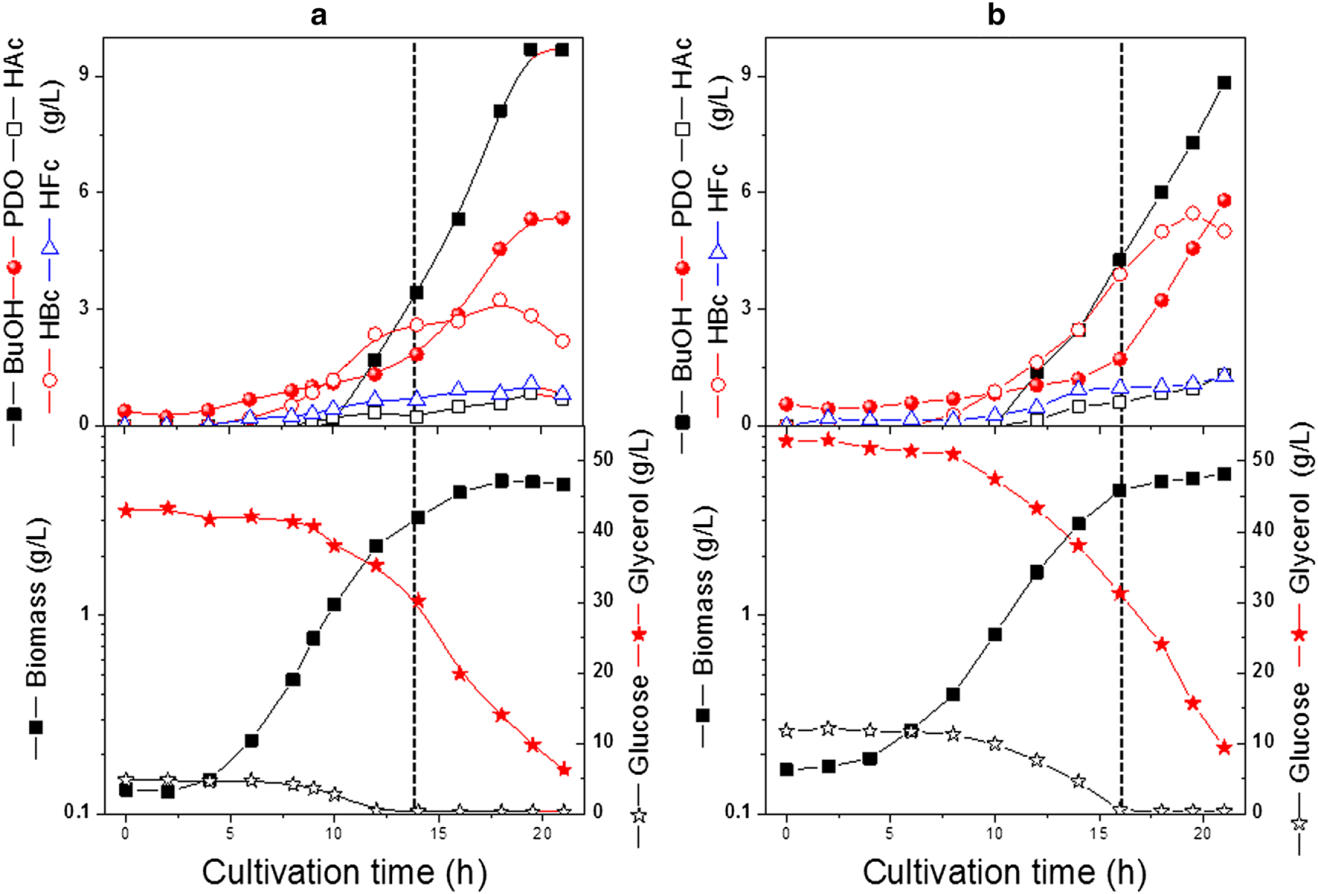
Growth, substrate consumption and product formation by *C. pasteurianum* with either 5 g/L (**a**) or 10 g/L (**b**) initial glucose concentration. Start of glucose limitation is indicated by *dotted line*. (*BuOH* n-butanol, *PDO* 1,3-propanediol, *HAc* acetate, *HBc* butyrate, *HFc* Formate)

Glycerol is a more reduced substrate than glucose, and thus for the same amount of carbon, twice as much NADH is generated as from glucose. The reducing equivalent excess provided by the conversion of glycerol to pyruvate must be oxidized through NADH uptake pathways. Previously it was reported that in *C. butyricum* grown on a mixture of glucose and glycerol, the glucose catabolism was mainly used to produce energy through the acetate–butyrate production, whereas glycerol was mainly used for the consumption of the reducing power via 1,3-PDO production [12]. Few studies have been reported for the growth and metabolism of *C. pasteurianum* in mixed substrate fermentation [10, 11]. In this work, the growth and product formation of *C. pasteurianum* were first studied in bioreactor cultures with the same and relatively high glycerol concentration (50 g/l) but varied initial glucose concentrations. An interesting phenomenon was observed that is related to the initial glucose concentration used. With 5 g/L (or less, data not shown) initial glucose concentration, cells continued to grow on glycerol after glucose exhaustion (Fig. 1a), whereas cells ceased growing upon glucose limitation, when the initial glucose concentration was 10 g/L, irrespective of the presence of glycerol (Fig. 1b). The concentrations of different products measured in these cultures are depicted in Fig. 1. The calculation of specific growth rate (µ) directly before and after the glucose limitation in the two cultures revealed that cells in the culture with 5 g/L glucose grew faster both before (µ = 0.37 vs. 0.29 h^−1^ in the culture with 10 g/L) and after the glucose limitation (µ = 0.15 vs. 0.03 h^−1^ in the culture with 10 g/L) (Table 1). Parallel to the higher growth rate at 5 g/L glucose, the specific glucose consumption rates were also higher compared to the culture with 10 g/L glucose, whereas the specific glycerol consumption rates before the glucose limitation were comparable between the two fermentations (Table 1).

**Table 1:** Comparison of key parameters of *C. pasteurianum* cultures grown on mono and dual substrates

	Dual substrate fermentation with			Mono-substrate fermentation	
	5 g/L glucose	10 g/L glucose	10 g/L glucose (GS)	Glucose	Glycerol
Butanol concentration at the onset of stationary phase (g/L)	8.01 ± 2.10	4.61 ± 1.50	2.23 ± 0.20	3.2 ± 0.40	14.08 ± 0.90
Total acids at the onset of stationary phase (g/L)	2.20 ± 0.15	4.50 ± 0.50	4.41 ± 0.40	25.3 ± 2.90	0.5 ± 0.12
Maximum biomass concentrations at the onset of glucose limitation (g/L)	2.13 ± 0.12	4.49 ± 0.28	3.97 ± 0.21	–	–
Maximum biomass concentrations in batch phase (g/L). (maximum biomass values after re-addition of glucose)	4.79 ± 0.12 (5.01 ± 0.08)	4.49 ± 0.28 (6.5 ± 0.21)	3.97 ± 0.21 (6.29 ± 0.15)	11.53 ± 0.57	4.88 ± 0.25
Growth rate before glucose limitation (h^−1^)	0.37 ± 0.15	0.29 ± 0.01	0.29 ± 0.03	0.25 ± 0.01^a^	0.23 ± 0.03^a^
Growth rate after glucose limitation (h^−1^)	0.15 ± 0.08	0.03 ± 0.01	0.02 ± 0.03	–	–
Specific uptake rate of glucose before glucose limitation (g/g/h)	0.91 ± 0.05	0.76 ± 0.07	0.72 ± 0.31	1.7 ± 0.21^a^	
Specific uptake rate of glycerol before glucose limitation (g/g/h)	1.45 ± 0.28	1.47 ± 0.11	1.62 ± 0.25	–	3.4 ± 0.35^a^

The values are average of triplicates and the standard deviations (in parentheses) are given

*GS* Fermentation with gas tripping, in situ butanol removal by gas stripping, *qs* Specific substrate consumption rate (g/g biomass/h)

^a^Values during the exponential growth phase of culture on single substrate, growth rate or qs in exponential phase with mono-substrate fermentation

### Physiological analysis

**Fig. 2:**
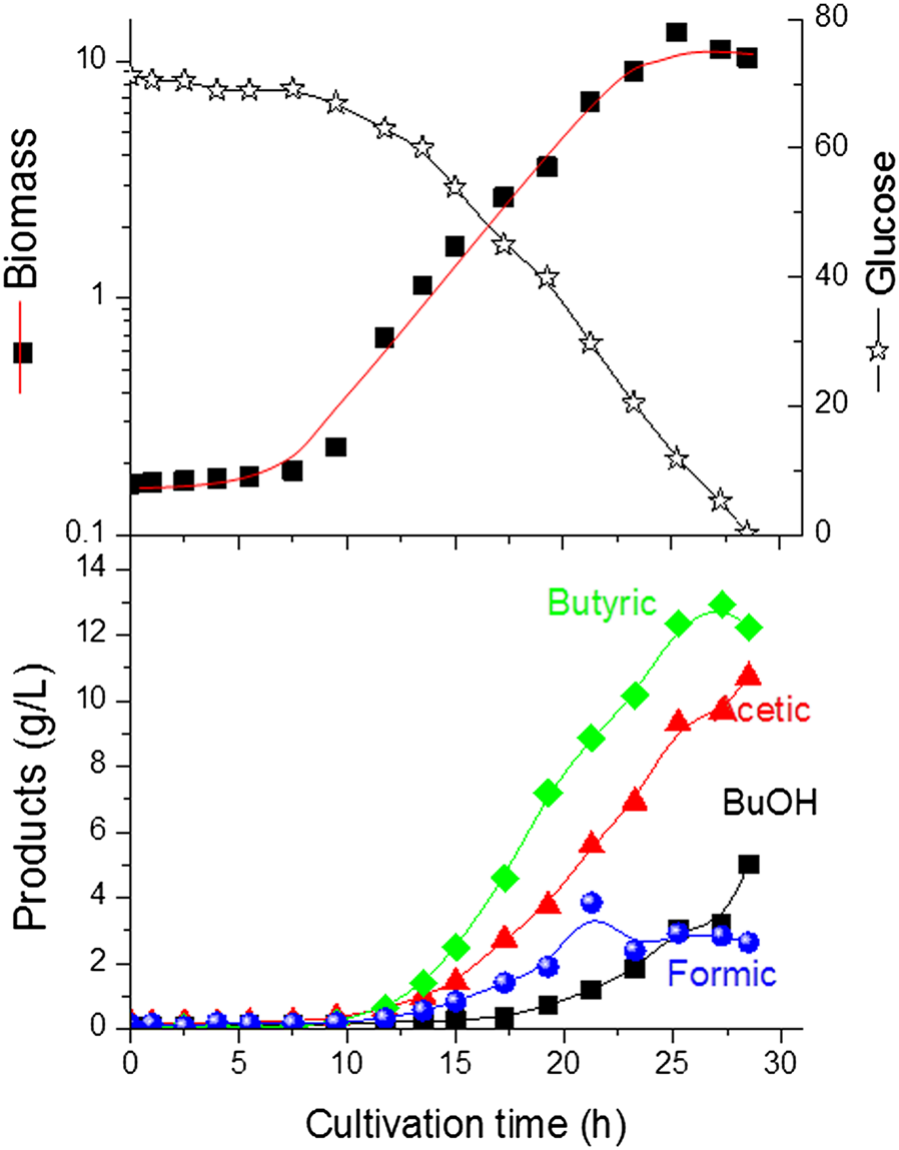
Growth and product formation of *C. pasteurianum* on glucose as the sole carbon source

As shown in Fig. 1, with increased initial glucose concentration in the medium, acids production, mainly butyric acid increased significantly. At the onset of glucose limitation, the concentration of butanol measured was less than 5 g/L in the two cultures with 5 and 10 g/L initial glucose concentration. Normally, in slightly acidic culture, organic acid and butanol can cause stresses to cells which mutually overlap, and may elicit a complex response in cells. However, the significant increase in acid production in the cultivation with 10 g/L initial glucose concentration (4.5 compared to 2.2 at 5 and 10 g/L glucose, respectively) cannot explain the cessation of cell growth, since the same strain grown on glucose as the sole carbon source can tolerate together up to 25 g/L of acetic and butyric acids (Fig. 2; Table 1). Furthermore, for the culture with 5 g/L initial glucose concentration, the cells continued to grow after the glucose limitation and entered the stationary phase first after butanol concentration reached about 10 g/L. This indicates that the growth cessation in the fermentation with 10 g/L initial glucose concentration was also not due to the toxicity of till then accumulated butanol of 4 g/L. It should be mentioned that in our previous study [10] with 20 g/L initial glucose concentration, a much higher butanol concentration (more than 11.5 g/L) was achieved at the onset of glucose limitation and that was the main reason for the stop of cell growth and glycerol uptake. To exclude the possibility of significant inhibitory effect on cell growth due to high intracellular concentration of n-butanol in the present study, similar experiments using in situ butanol removal by gas stripping were carried out at both 5 and 10 g/L initial glucose concentration [13]. But the same cell growth behavior was also observed (Additional file 1: Figure S1).

**Fig. 3:**
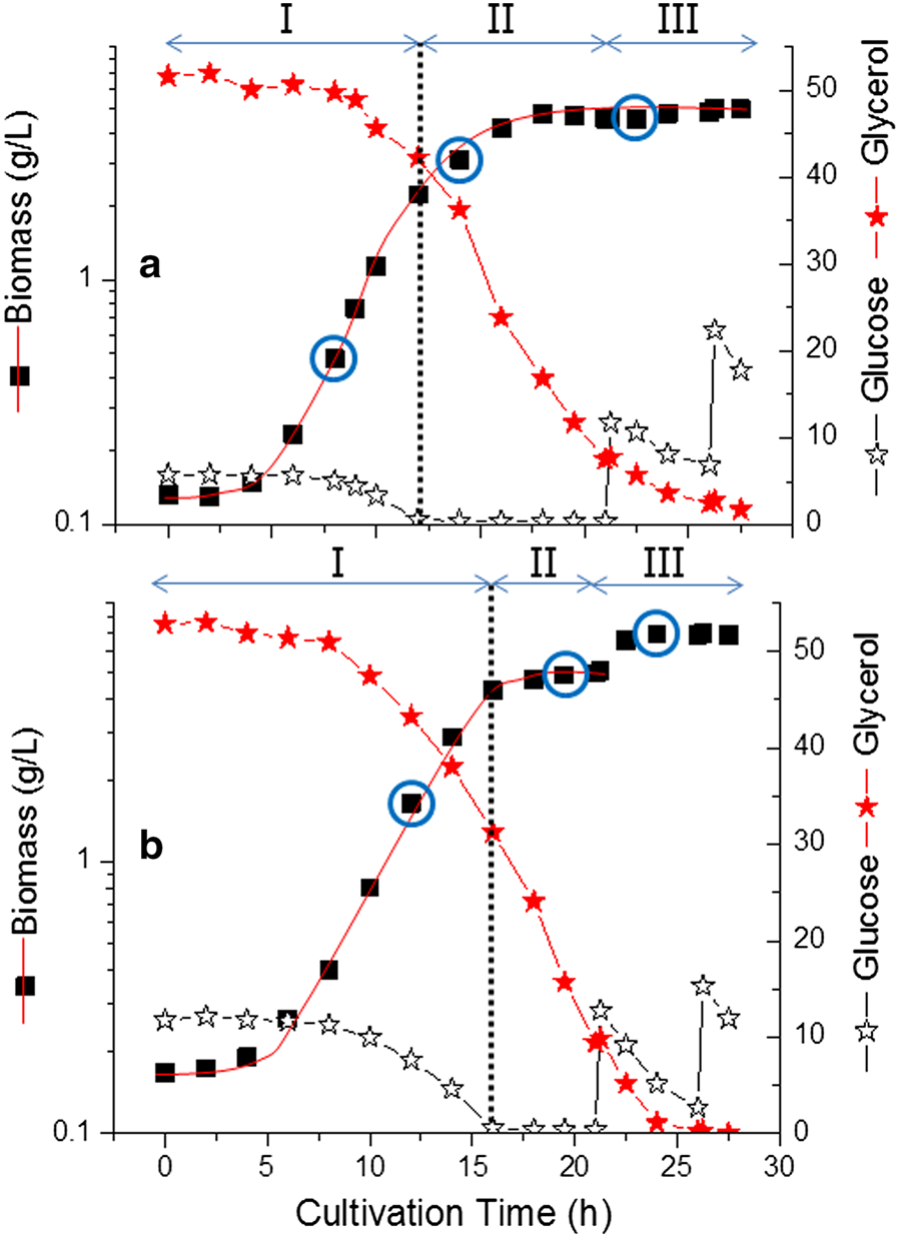
Time profile of dual substrate fermentation by C. pasteurianum with either 5 g/L (**a**) or 10 g/L (**b**) initial glucose concentration. I, II and III show the different phases where samples were taken for comparative proteomic analyses

Normally in a culture entering the stationary phase, the metabolism of the bacterial cells will change. As recently reported for *C. pasteurianum* by Kolek et al. [14], the fatty acid composition of the cell membrane may also change. The ratio of saturated to unsaturated fatty acids has been used as an indirect indicator of membrane fluidity. Kolek et al. [14] reported that a higher butanol concentration was the main trigger for the change in the membrane composition in *C. pasteurianum* [14]. In this study, the membrane fluidity was also studied in response to glucose limitation in the mixed-substrate fermentations with 10 and 5 g/L initial glucose concentrations, respectively. Fatty acid composition of the cell membrane was measured for cell samples taken at mid logarithmic phase when both substrates were in excess and at stationary phase (Fig. 1). Palmitic acid (C16:0), stearic acid (C18:0) and heneicosanoic acid (C21:0) as saturated fatty acids and palmitoleic acid (C16:1) and oleic acid (C18:1) as unsaturated fatty acids were the major fatty acids detected in the cell membrane of *C. pasteurianum*. Despite the differences in the growth phases, the ratio of saturated to unsaturated fatty acids remained constant at 4.1 ± 0.3 before and after glucose limitation for the 10 g glucose/L culture, whereas a slight increase was noticed for the culture with 5 g glucose/L (ration of 5.3 ± 0.8 in the mid exponential phase and 6.1 ± 1.1 at the stationary phase). This indicates that the membrane fluidity of cells was not significantly affected by any stresses possibly caused by accumulated butanol or glucose limitation in the culture with 10 g/L initial glucose concentration. Moreover, the re-addition of glucose shortly after the onset of stationary phase in the 10 g/L initial glucose concentration revived the growth and the biomass production increased from 5.1 to 6.9 g/L (Fig. 3). In contrast, the re-addition of glucose to stationary phase culture with 5 g/L initial glucose concentration did not revive cell growth. This phenomenon was reproducibly observed in three independent fermentation experiments carried out at different times. The growth and fermentation patterns of *C. pasteurianum* DSM 525 with dual substrates from the triplicate experiments are summarized in Table 1.

### Comparative proteome analysis

To understand the regulation of cell growth at a more systems level, proteomic analysis was carried out. As shown in Fig. 3, three major phases of cell growth found to be mostly related were chosen for proteomic analysis: I exponential growth phase, in which cells used both substrates; II glucose limited phase and III after glucose addition. Comparison was made between two fermentations with 5 and 10 g/L initial glucose concentration (Table 2; Additional file 1: Table S1, S2), respectively, and among the different growth phases in the fermentation with 10 g/L initial glucose concentration (Additional file 1: Table S1).

**Table 2:** List of proteins whose levels showed at least twofold change during the course of fermentation with 5 and 10 g/L initial glucose without gas stripping, before and after glucose limitation

Gene name	Function or description	Fold change (II/I) (5 g/L)	Fold change (II/I) (10 g/L)
F502_14770	Serine protein kinase	18.0, 12.8, 4.7	12.3, 5.4, 2.7
F502_06242	Chaperonin GroEL	–	7.5, 6.4,2.0
F502_00655	Peptidoglycan-binding protein LysM	12.6	6.1
F502_18092	Stage V sporulation protein T	12.2	–
F502_15080	Rubrerythrin	2.9	5.5
F502_07198	Single-stranded DNA-binding protein	7.5	–
F502_16610	Glycolate oxidase	2.7	5.5
F502_16565	Nitrogen regulatory protein P-II	6.2	5.3
F502_03342	Pyruvate phosphate dikinase	4.4, 4.2	4.9, 3,0
F502_04232	Stage IV sporulation protein A	4.1, 3.3	4.6, 2.6
F502_18651	NADP-dependent glyceraldehyde-3-phosphate dehydrogenase	4.3, 2.6	4.4
F502_14915	Alpha-glucosidase	3.2	–
F502_03937	Gene_glgA glycogen synthase	3	–
F502_06067	Enolase	2.1	3.8, 2.0
F502_14780	SpoVR family protein	3.2, 2.0	3.7, 2
F502_06247	Co-chaperonin GroES	–	3.5
F502_09238	Rubredoxin/flavodoxin/oxidoreductase	3.4, 3.1	3.2, 3.0
F502_05347	Putative phosphate starvation-inducible protein PhoH	4.2	3
F502_04697	Phosphocarrier protein (HPr)	3.2	2.6
F502_03987	Peptidase	7.4, 4.8, 4.0	2.4, 1.6
F502_15100	Oligoendopeptidase F	–	2.4
F502_06447	Bifunctional acetaldehyde-CoA/alcohol dehydrogenase	2.2	–
F502_09058	Thiamine pyrophosphate protein central region	2.6	–
F502_14060	Acetoin reductase	–	2.3
F502_12878	Desulfoferrodoxin	–	2.2
F502_00410	Isoleucyl-tRNA ligase	2.3	–
F502_05157	dTDP-4-dehydrorhamnose reductase	2.8	–
F502_19151	Hypothetical potein	2.6	–
F502_18446	Chaperone protein clpb	–	2.1
F502_04537	30S Ribosomal protein S2	–	−4.5
F502_02505	Biotin synthase	−2.2	−4.1
F502_11976	Pyruvate carboxylase	–	−4
F502_12326	Transcription accessory protein TEX, RNA-binding protein containing S1 domain	−3.6	–
F502_07798	Flagellin	−2.9	–
F502_07498	Formiminotransferase-cyclodeaminase	−2.9, −5.4	–
F502_09488	Hydratase	−2.8	–
F502_18706	Prolyl-tRNA ligase	−2.7	–
F502_07578	Pyridoxal biosynthesis lyase PdxS	−2.6	−3.2
F502_04127	Cell division protein FtsZ	−2.6	−3.0, −2.8
F502_08238	Cell division protein DivIVA	–	−2.7
F502_07413	DTPD-d-glucose 4,6 -dehydratase	−2.2	−2.5
F502_00710	Gene_pyrG CTP synthetase	−2.2	−2.3
F502_05017	NifU related domain containing protein	–	−2.1
F502_04707	Adenylosuccinate lyase	–	−2
F502_10588	Ferritin 50S	−3.5	−2
F502_18843	Ribosomal protein L7/L12	–	−2
F502_18848	DNA-directed RNA polymerase subunit beta	–	−2
F502_18292/18287	Hydrogene dehydrogenase/hydrogenase-1	–	−3.0, −2.5
F502_06232	Gene_guaA GMP synthase	−2	–
F502_07643	Pyruvate:ferredoxin (flavodoxin) oxidoreductase	−2.1, −2.3, −3.0	–
F502_03482	Dihydroxy-acid dehydratase	−2.4	−1.9
F502_15435	Fructokinase	−2.1	–
F502_19674	Aspartate kinase	−2.2	–
F502_12231	Hypothetical protein	−2.3	–
F502_19118	Phosphoenolpyruvate-protein phosphotransferase	−2.3	−1.8
F502_06272	Ferredoxin-NADP(+) reductase subunit alpha	−2.9	–
F502_14710	Hypothetical protein	−2.3	–
F502_12241	Hypothetical protein	−2.3	–

**Fig. 4:**
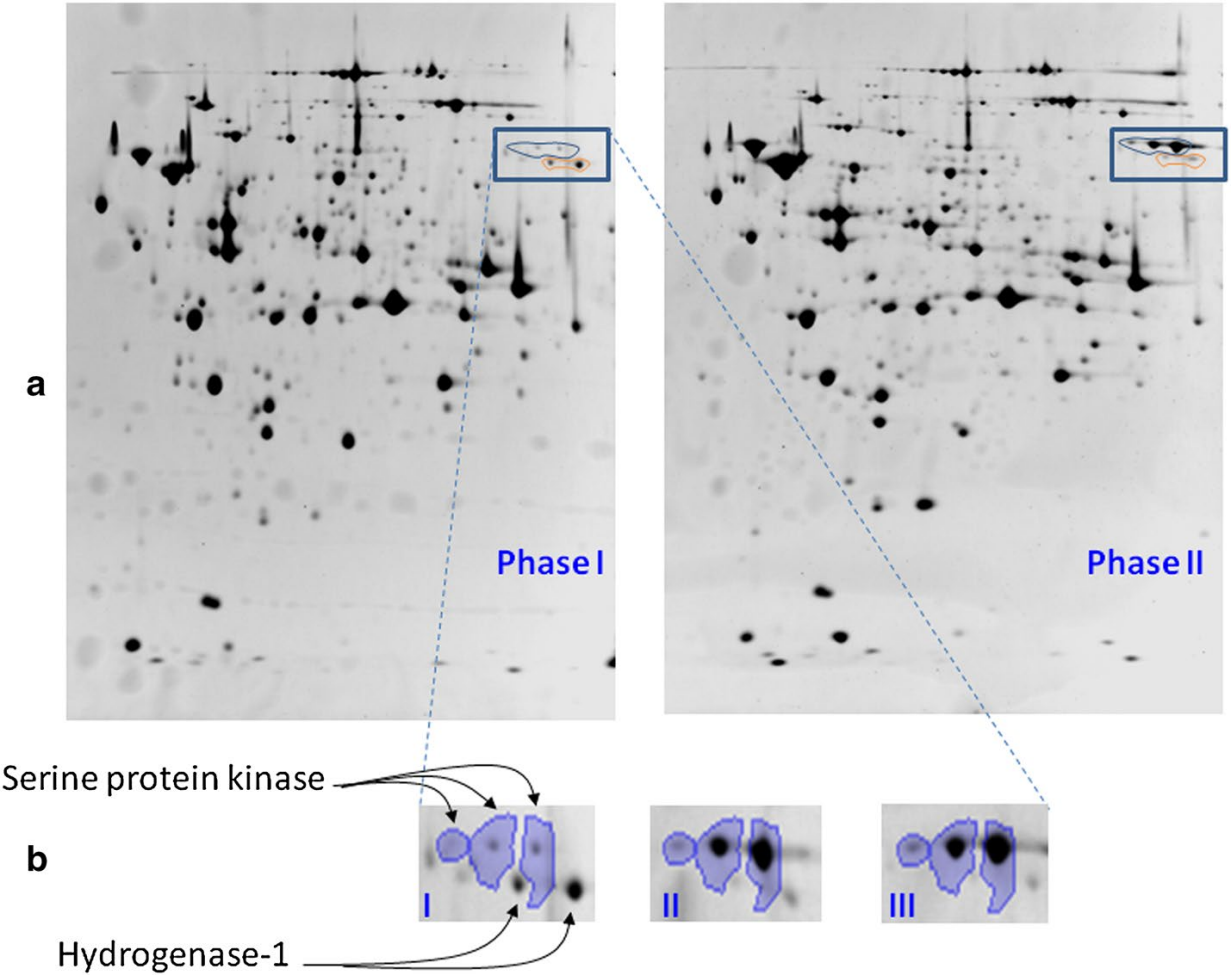
2-DE maps of the proteomes of *C. pasteurianum* grown on a blend of glucose and glycerol before (Phase I) and after glucose limitation (Phase II) (**a**). Magnified 2-DE image showing the differential expression of serine protein kinase (*3 spots*) and hydrogenase (*2 spots*) between the 3 phases in the fermentation of 10 g/L initial glucose concentration (**b**)

Rappert et al. [15] published the draft genome sequence of *C.pasteurianum* DSMZ 525. Using this genomic data as a basis we established a comprehensive two dimensional electrophoresis 2-DE reference map of the cytoplasmic proteins of *C. pasteurianum* DSMZ 525 and used this long-proven proteome technique to explain the dependence of growth on the presence of glucose in the dual substrate fermentations with 5 and 10 g/L initial glucose concentrations. To our knowledge, this is the first proteomic study on *C. pasteurianum*. Over 500 protein spots were resolved on the 2-D gels. In total, 55 and 48 protein spots showing significant expression changes (Anova-P ≤ 0.05, fold change ≥2) were identified in the fermentations with 5 and 10 g/L initial glucose concentration (Fig. 3), respectively. In the fermentation with 10 g/L initial glucose concentration, expression levels of 30 and 18 proteins were identified to be up- and down-regulated, respectively, in phase II (glucose limited phase) compared to phase I (exponential growth phase) (Table 2; Fig. 4), but only a few of them showed significant changes after glucose addition in phase III compared to phase II (Additional file 1: Table S1). In the fermentation with 5 g/L initial glucose concentration, 32 and 23 proteins were up- and down-regulated, respectively, in phase II compared to phase I (Table 2; Fig. 4).

As shown in Table 2, in response to glucose limitation and with glycerol as the remaining carbon source (phase II), several proteins, namely pyruvate phosphate dikinase (PPdK), NADP-dependent glyceraldehyde-3-phosphate dehydrogenase (GAPDH) and enolase (Eno), involved in the interconversion of the intermediates of the glycolysis/gluconeogenesis pathway were found to be up-regulated in both fermentations. Pyruvate phosphate dikinase, known to catalyze the interconversion between pyruvate and PEP has been found to be induced on the transfer of glucose-grown cells of *Acetobacter xylinum* to succinate- or pyruvate-containing media [16], serving thereby a gluconeogenic function. However, in *C. symbiosum*, PPdK substitutes for the absent pyruvate kinase and thus fulfils a glycolytic function [17]. In *Thermoproteus tenax* PPdK shows a bidirectional activity with a preference for the catabolic reaction, and it is suggested that PPdK serve as a ‘stand-by’ enzyme and thus allows for quick adaptation to changing intracellular conditions [18]. The NADP-dependent GAPDH is a multifunctional enzyme capable of catalyzing among others the interconversion between glyceraldehyde-3-phosphate and 3-phosphoglycerate as well as the conversion of 1,3-bisphosphoglycerate to glyceraldehydes-3-phosphate. Whether these enzymes serve a glycolytic or gluconeogenic function is not clear, however, their up-regulated expression highlighted their importance in the central carbon metabolism of *C. pasteurianum* in response to glucose limitation conditions, and they might all serve as ‘stand-by’ enzymes for a rapid adjustment of the central metabolic fluxes to changes in nutrient availability.

Proteins which also showed increased levels in phase II in both fermentations also include those involved predominantly in stress sensing and stress responses. Examples of these proteins include serine protein kinase, rubrerythrin and rubredoxin/flavodoxin/oxidoreductase related to oxidative stress or heat shock, sporulation proteins, like stage IV sporulation protein A and SpoVR family protein, the phosphate starvation-inducible PhoH family protein, and the signaling protein nitrogen regulatory protein P-II involved in the regulation of nitrogen metabolism.

In fact, serine protein kinase was one of the mostly up-regulated proteins after glucose limitation (Fig. 4). Compared to phase I, the three identified spots of serine protein kinase were 12.3-, 5.4- and 2.7-fold up-regulated in phase II in the fermentation with 10 g/L initial glucose concentration. Expression of serine protein kinase has been reported to be positively controlled by guanosine pentaphosphate [(p)ppGpp] and involved in (p)ppGpp-induced stringent response upon nutritional deprivation. For example, it is strongly up-regulated during stationary phase and involved in metabolic adaptation in *Rhizobium etli* [19] or in sporulation of *Bacillus subtilis* [20]. Therefore, the strong upregulation clearly indicates the stringent response and the metabolic switching of *C. pasteurianum* upon glucose depletion. After the re-addition of glucose (phase III) no further change on the expression levels of these protein spots was observed (Additional file 1: Table S1). The up-regulation of serine protein kinase was even stronger in the fermentation with 5 g/L L initial glucose concentration after entering glucose limitation. Compared to phase I the expression levels of three spots showed 18.0-, 12.8- and 4.7-fold of increases in phase II, despite the fact that cells had not yet entered the stationary phase but were still growing on glycerol in phase II. Therefore, the (p)ppGpp-induced stringent response shown by serine protein kinase was triggered by glucose limitation, though limitation of other nutrients could not be totally ruled out. This glucose limitation-caused stringent response might further trigger a set of cellular reactions to glucose limitation, including the expression of sporulation proteins. Indeed, in the fermentation with 5 g/L initial glucose concentration, except the above mentioned two spore proteins, an additional spore protein, the stage V sporulation protein T, was found to be strongly up-regulated by 12.2-fold (Table 2).

**Fig. 5:**
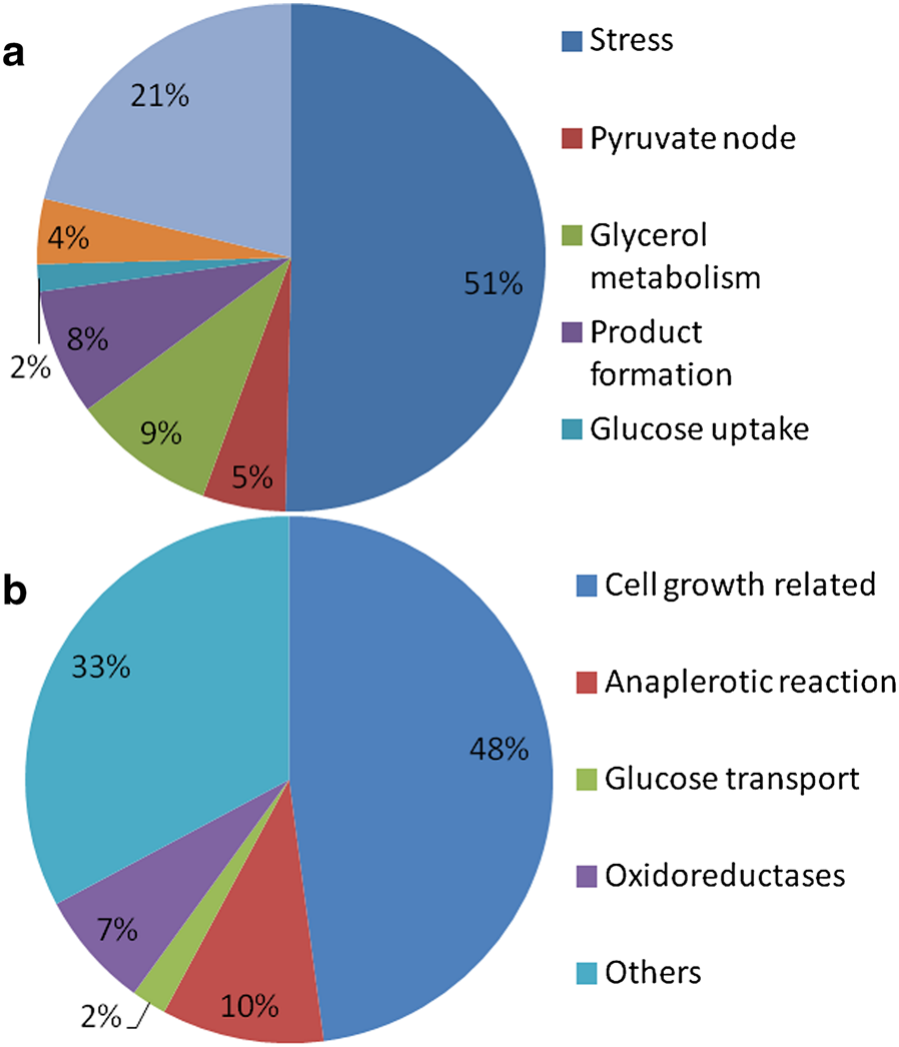
Functional classification of proteins whose level increased (**a**) or decreased (**b**) in *C. pasteurianum* DSMZ 525 cells grown in dual substrate fermentation (10 g/L initial glucose concentration) before and after glucose limitation (I and II growth phases)

Interestingly, chaperones, which were found to be highly up-regulated upon glucose limitation in the culture with 10 g/L initial glucose concentration (GroEL, GroES, ClpB) were not among the significantly up-regulated proteins after glucose limitation in the culture with 5 g/L initial glucose concentration (Table 2). Chaperones are proteins known to play essential role in the folding and/or assembly of proteins, and were found to be expressed in response to stresses by many bacteria [21]. In fact, after glucose limitation in the fermentation with 10 g/L initial glucose concentration, more than 50 % of the over-expressed proteins were associated with stress response (Fig. 5).

The decrease in the specific growth rate observed after glucose limitation at both initial glucose concentrations (Table 1), may explain the down regulation of some cell growth associated proteins, such as cell division proteins FtsZ and DivIVA, pyridoxal biosynthesis lyase PdxS for the synthesis of vitamin B6). Two ribosomal proteins, 30S ribosomal protein S2 and 50S ribosomal protein L7/L12, were found to be down-regulated by −4.5- and −2-fold, respectively, but only in the fermentation with 10 g/L initial glucose concentration. Previous studies showed the linear correlation of the ribosomes concentration in bacterial cells with their growth rate, the ribosomes concentration is drastically reduced compared to logarithmic growth phase [22]. Thus, the reduced expression levels of the ribosomal proteins could correlate to the cessation of growth at phase II in the fermentation with 10 g/L initial glucose concentration, whereas to the sampling time point at phase II in the fermentation with 5 g/L initial glucose concentration, cells did not enter stationary phase after glucose limitation but continued to grow on glycerol.

**Fig. 6:**
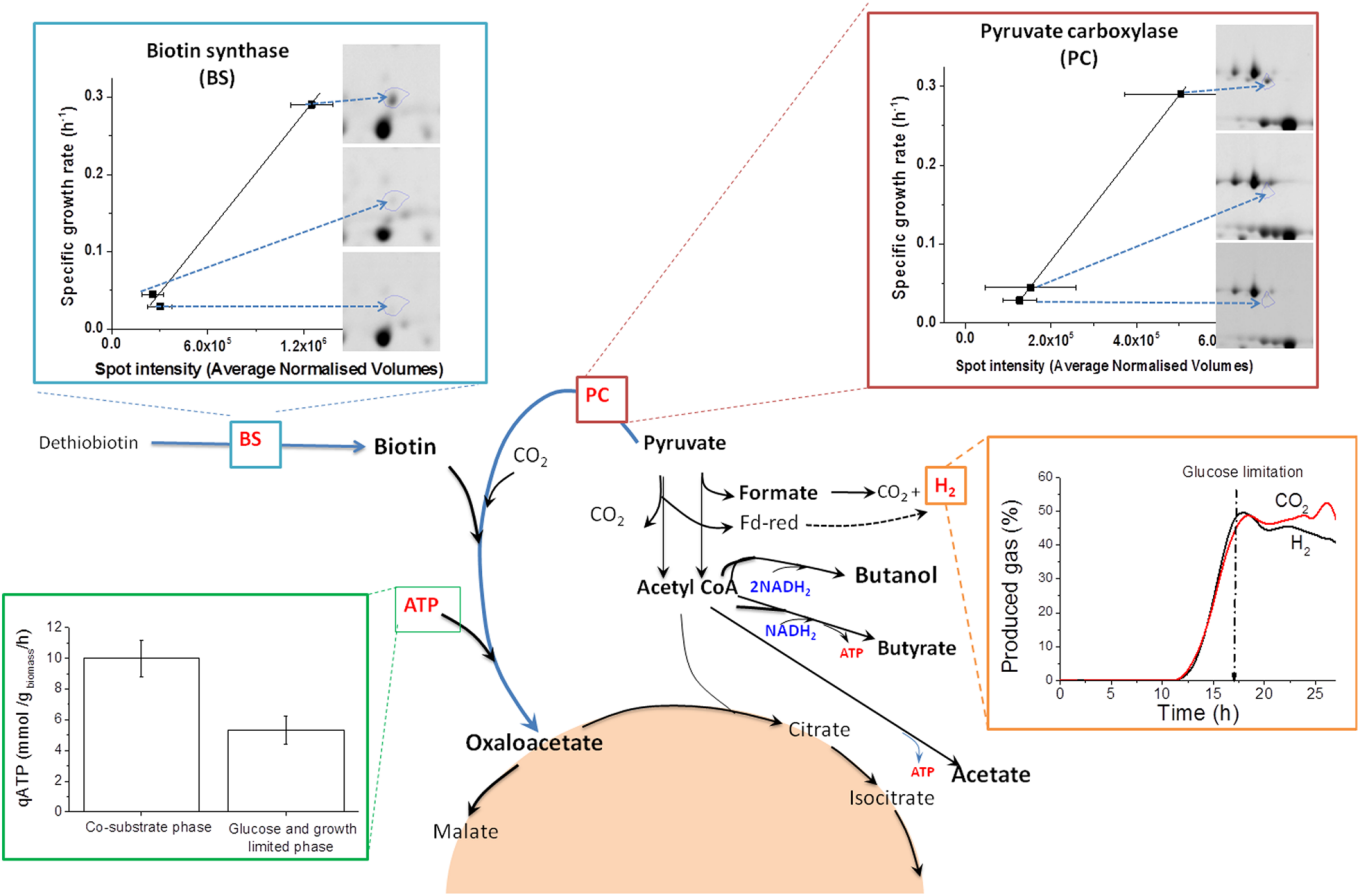
Correlation of the specific growth rate of *C. pasteurianum* with the expression level of pyruvate carboxylase and biotin sythase, enzymes necessary for the anaplerotic formation of oxaloacetate. The decrease in the ATP production rate and hydrogen production triggered by glucose limitation is also shown

A significant difference between the two fermentation with different initial glucose concentrations was the changes in the expression level of the anaepleoretic pyruvate carboxylase (PC), which catalyzes the formation of oxaloacetate from pyruvate (Table 2). Only with 10 g/L initial glucose concentration, PC was found to be down-regulated in the phase II in the absence of glucose. Biotin synthase, which is also required as a cofactor for the carboxylation of pyruvate catalyzed by PC was found to be highly down regulated in phase II as well (−4.1-fold compared to −2.2-fold at 5 g/L initial glucose concentration). PC serves an anaplerotic role in the formation of oxaloacetate which acts as a direct precursor for the biosynthesis of aspartate/asparagine, lysine, threonine, and β-alanine (pantothenate) and indirectly for the biosynthesis of arginine, histidine, purine, nicotinate, and nicotinamide. In the anaerobic pathogenic bacterium *Listeria monocytogenes*, PC defective mutant was unable to multiply in a defined medium with glucose or glycerol as carbon source [23]. Interestingly, in the fermentation with 10 g/L initial glucose concentration (Fig. 3b), after re-addition of glucose (phase III), and compared to the glucose limited phase (phase II) a slight up-regulation of PC (1.2-fold, Anova-P = 0.014) was noticed (Additional file 1: Table S1), compared to the glucose limited phase (phase II). This up-regulation of PC coincided with the increase in growth rate observed after glucose re-addition (1.3-fold at an Anova-P = of 0.014). In fact, a good correlation between the fold decreases in the expression levels of both PC and biotin synthase and the growth rate decrease could be established in *C. pasteurianum* cultures, as shown in Fig. 6. At 5 g/L initial glucose concentration, where the bacterial growth continued even after glucose limitation (Fig. 1), the expression level of PC was not found to be significant changed (Table 2).

In general, H_2_ is generated in Clostridia in a ferrodoxin-dependent reaction, and till recently the reactions used to regenerate the reduced ferrodoxin in vivo were not known [24]. In *C. ljungdahli*, it has been recently shown that Rnf complex in the cell membrane is a proton-translocating ferredoxin:NAD+ oxidoreductase which contributes to ATP synthesis by an proton-translocating ATPase [25]. From the genome of *C. pasteunianum* DSM 525 studied in this work, *C. pasteunianum* DSM 525 possesses an arsenal of hydrogenases but not all the components comprising the Rnf complex. Among the hydrogenases are three ferrodoxin-dependent hydrogenases encoded by the genes F502_14390, F502_17487 and F502_13920, as well as two non-ferrodoxin-dependent hydrogenases, namely the hydrogene dehydorgenase and the hydrogenase-1, which are 100 % identical in protein sequence but encoded by two adjacent genes F502_18292 and F502_18287, respectively (http://www.biocyc.org/organism-summary?object=CPAS1262449). Interestingly, none of the ferrodoxin-dependent hydrogenases were among the significantly regulated proteins, whereas the non-ferrodoxin-dependent hydrogenases (hydrogene dehydorgenase and/or hydrogenase-1) were found to be highly down-regulated after glucose limitation, however, only in the fermentation with 10 g/l initial glucose concentration (Table 2). Hydrogenase-1 is annotated as a menaquinone-dependent proton-translocating enzyme in *C. pasteurianum* DSM 525 by translocating a pair of protons outside the cell membrane with concomitant consumption of H_2_ present outside the cell membrane (http://www.biocyc.org/gene?orgid=CPAS1262449&id=G10RN-3668#). Therefore, hydrogenase-1 might be involved in the ATP synthesis and its down-regulation after glucose limitation might negatively affect the ATP production. The shift from dual substrate fermentation with glucose and glycerol to mono substrate fermentation with glycerol, a more reduced substrate than glucose, will certainly alter cellular response to achieve a new redox balance. The differences in the regulation of the menaquinone-dependent proton-translocating enzyme between the two fermentations with different initial glucose concentrations are interesting but still elusive and deserve further investigation.

### Bioeneregtics and cellular metabolism

ATP is the energy currency of the cell, providing energy for cell growth and maintenance. It also serves as a substrate for RNA synthesis, and regulates a variety of biological processes [26]. Intracellular ATP concentration is also important for the in vivo regulation of many metabolic pathways at the enzyme level [27]. In *C. pasteurianum*, substrate level phosphorylation and ATP formation are generally coupled to acids production. In fact, the relatively higher acids production with glucose as C-source, and hence the increase in the ATP production rate, together with the lower butanol formation, may explain the relatively high biomass concentration obtained with glucose (Fig. 2; Table 1). Additionally, with glucose as the sole C-source, the relative tolerance towards butanol inhibition was enhanced (compared to glycerol as the sole C-source, Additional file 1: Figure S2). To examine whether the bioenergetics status of the cells after glucose limitation was the reason for cell growth cessation at 10 g/L initial glucose concentration, ATP formation rate after glucose limitation at both initial glucose concentrations were here studied. Compared to the dual substrate utilization (Phase I), the calculated specific ATP production rate (mmol/g_biomass_/h) decreased by about 45 and 30 % after glucose limitation (Phase II) for the two cultures with 5 and 10 g/L initial glucose concentration, respectively, and decreased further in phase III by about 55 and 40 %, respectively (Fig. 6). Obviously, the decrease in the ATP production owing to glucose limitation cannot alone explain the phenomenon observed in the dual substrate fermentation of *C. pasteurianum* caused by the difference in initial glucose concentration, but it may contribute to the inhibition of the pyruvate carboxylase reaction and the overall cellular responses to glucose limitation.

**Fig. 7:**
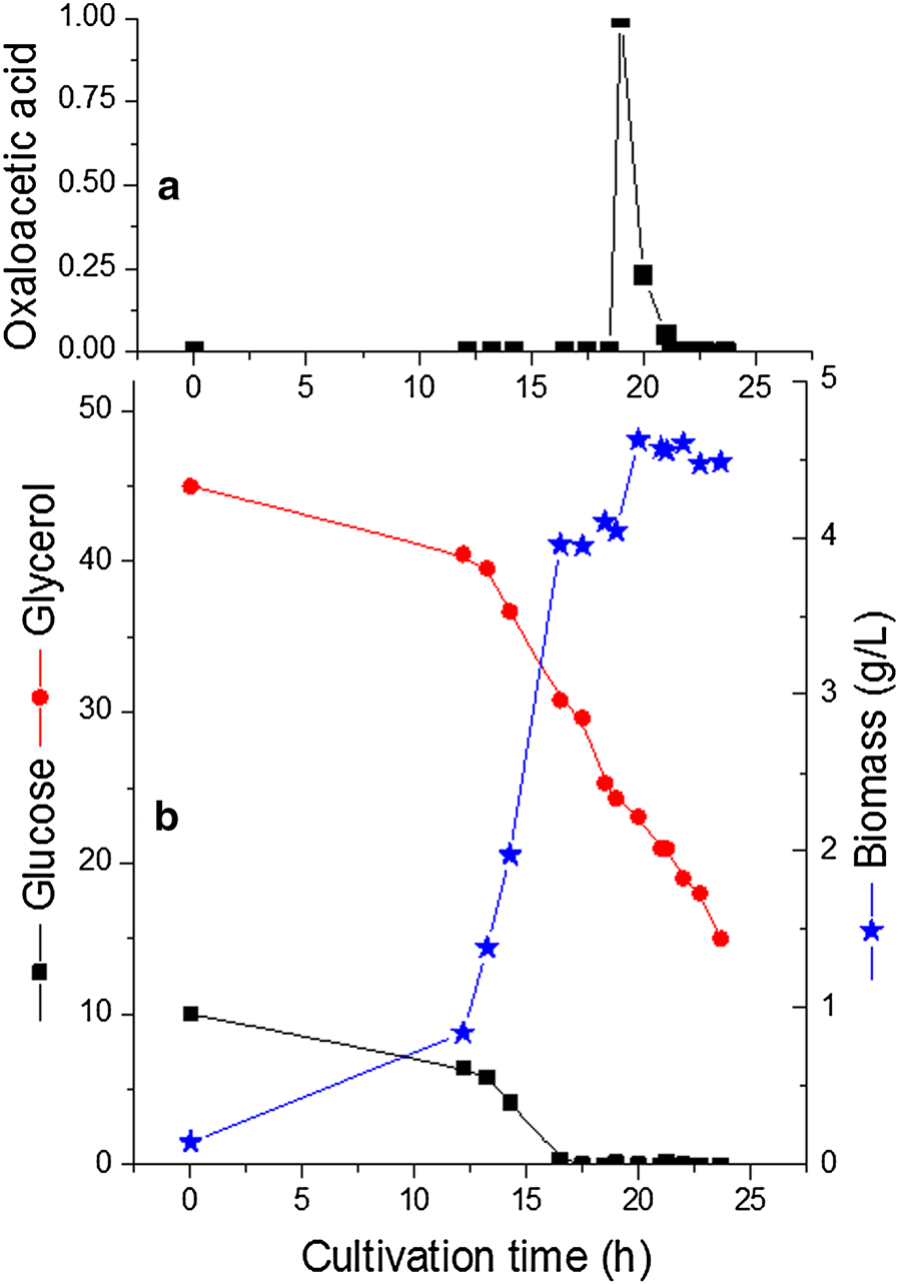
Effect of oxaloacetate addition on the growth of* C. pasteurianum* in glucose limited dual substrate fermentation

Interestingly, the addition of biotin to the glucose limited phase did not retrieve the growth in glucose limited medium. On the other hand, oxaloacetate addition to the glucose limited mixed-substrate fermentation resumed the growth and an increase of the OD from 12 to 13.8 was recorded (Fig. 7). Obviously, the growth requirement and metabolism on blend or on glycerol as mono-substrate are different, and the cellular response redistributes the composition of the fermentation end products to achieve a balance of reducing power. Indeed, in the experiment with 10 g/L initial glucose concentration, the hydrogen production rate decreased steadily whereas the CO_2_ production rate remained constant after glucose limitation (Fig. 6). This explains the continuous production of both butanol and 1,3-PDO after glucose and growth limitation with 10 g/L initial glucose concentration, since they are correlated to the consumption of NADH. The increase in the in vivo activity of hydrogenases with dual substrates compared to glycerol fermentation were reported previously for *C. acetobutylicum* [27].

Carbon sources affect the kinetics of *C. pasteurianum* growth through a cellular response which distributes the product formation to achieve a balance of oxidation and reduction reactions. In a medium with excess carbon source, two distinct conditions exist under which the growth may cease in batch culture of *C. pasteurianum:* (i) butanol overproduction which challenge the cells to increase the percentage of saturated fatty acids and the formation of more rigid or stable membranes in the stationary phase to counteract n-butanol fluidization [28] and (ii) metabolic shift after glucose limitation in the dual substrate fermentation which leads to abrupt decrease in acid production and the down regulation of pyruvate carboxylase. The analysis of fatty acids performed for samples before and after glucose limitation at 10 g/L initial glucose concentration indicated no significant difference in the saturated to unsaturated ratio, which also explain the revival of growth after either glucose or oxaloacetate addition. Our working hypothesis is thus that the growth limitation despite the presence of excess glycerol is partly due to the lack of precursor supply important for growth and to a less extent a decrease in cell energy balance.

## Conclusion

To develop a commercial process for the production of n-butanol by *C. pasteurianum*, it is necessary to better understand the primary metabolism and its regulation. Whereas glucose utilization supports biomass and acid production, glycerol utilization leads to significantly lower biomass formation but higher alcohol productions. The highest butanol production by *C. pasteurianum* was recorded in fermentations using a dual substrate of glucose and glycerol rather than in any of the mono-substrate fermentations. However, in the presence of excess amount of glycerol, the initial glucose concentration affects the cell growth after glucose limitation. Compared to the fermentation with 5 g/L initial glucose concentration, glucose limitation in the fermentation with 10 g/L initial glucose concentration leads to cease of cell growth even in the presence of excess glycerol in the medium, and hence affects negatively the butanol production. The physiological analyses in this work indicated that the cessation of cell growth is not primarily due to n-butanol toxicity. Moreover, cell membrane fluidity as indicated by fatty acid compositions did not change significantly before and after glucose limitation. The, to our best knowledge, first proteomic analysis of *C. pasteurianum* has provided some clues on the metabolic responses of *C. pasteurianum* to the changing growth conditions. Among others, the proteomic analysis has revealed the down-regulation of pyruvate carboxylase and biotin synthase as one of the major cellular responses which limit the anaplerotic formation of oxaloacetate and consequently the cell growth. Addition of oxaloacetate to the glucose limited culture has revived cell growth and thus confirms the limited availability of this intermediate as a key determinate of growth of *C. pasteurianum*. Thus, in order to improve the growth of *C. pasteurianum* and the productivity of n-butanol and eventually also other products from this bacterium efforts should be made to ensure the availability of glucose and oxaloacetate, i.e., through fed-batch fermentation or overexpression of genes related to the formation of oxaloacetate, especially pyruvate carboxylase and biotin synthase.

## Methods

### Bacteria strain, culture medium and growth conditions

*Clostridium pasteurianum* DSMZ 525 was cultivated anaerobically at 35 °C without shaking. The strain was maintained in Reinforced Clostridial Medium (RCM, Oxoid Deutschland GmbH, Wesel, Germany) and preserved using glycerin 20 % (v/v) at −80 °C.

RCM medium inoculated from a cryoculture was left to grow at 35 °C for 18–20 h and then used as inocula for the production medium. The standard production medium for batch cultures contained the following ingredients in 1 L of distilled water (modified from Biebl, [29]). Glycerol, varied; glucose, varied; K_2_HPO_4_, 0.5; KH_2_PO_4_, 0.5, MgSO_4_·7H_2_0, 0.2 g; (NH_4_)_2_SO_4_, 3 g; CaCl_2_ 2H_2_O, 0.02 g, FeSO_4_·7H_2_O, 0.01 g; cysteine HCL, 0.3; resarzurin, 0,005; trace element solution SL7, 2 mL and 1 g yeast extract.

Batch cultivations were carried out in a pH-controlled 2 L stirred tank bioreactor (bioengineering) with a working volume of 1.5 L. After sterilization, the medium in the fermenter was flushed with sterile O_2_-free N_2_ until room temperature was reached. Filter sterile cystein HCl and FeSO_4_ solution was then added and inoculated immediately. Glucose was autoclaved separately. Flushing with nitrogen was stopped after inoculation and the bacteria were grown under their own produced gases. The pH was adjusted to six by the automatic addition of 5 N KOH. Carbon dioxide and hydrogen signals were measured online by CO_2_ and H_2_ sensors connected in series according to the manufacturers instructions (BluSens GmBH, Germany).

To determine the effect of carbon source on the relative tolerance toward butanol, anaerobic bottles with production media containing either glucose or glycerol as the sole C-source and with different concentration of butanol were incubated and the initial growth rate after 8 h were calculated (equation below).

Since n-butanol concentration above about 5 g/L can negatively affect the bacterial metabolism, batch fermentation with mixed substrates and in situ removal of butanol by gas stripping was done according to Jensen et al. [30]. Briefly, own produced fermentation gases (mainly CO_2_ and H_2_) collected in the bioreactor headspace are pumped at a flow rate of 7 VVM through the fermentation broth and then to a condenser cooled at 1 °C (with a cooling surface of 0.34 m^2^). Condensates containing mainly butanol and water are collected in a cooled bottom separate vessel.

### Analytical methods

Cell concentration was measured optically, at 600 nm and correlated with cell dry weight determined directly. The concentrations of glucose, glycerol, butanol, 1,3 propanediol, ethanol, acetic, butyric, formic and lactic acids in supernatant were determined by HPLC using an Aminex HPX-87H column (300 × 7.8 mm) and the detection was assessed by refractive index and ultraviolet detectors. The operating conditions were as follows: mobile phase, H_2_SO_4_ 0.005 M; flow rate, 0.6 ml min^−1^; temperature, 60 °C.

For the determination of the cell wall fatty acid composition, GC analysis of the fatty acid methyl esters was performed as previously reported [31] with a Varian 3900 gas chromatograph equipped with a flame ionization detector (FID) and a TR-FAME column (Thermo Scientific, Germany, 50 m × 0.22 mm × 0.25 µm).

### Proteomic analysis

Comparative proteomic analysis was carried out by separating intracellular proteins using a two-dimensional gel electrophoretic method (2-DE) established in our lab [32]. Briefly, cells samples re-suspended in a lysis/rehydration buffer containing 7 M urea, 2 M thiourea, 4 %w/v CHAPS, 100 mM DTT, 0.5 % IPG buffer 3–10 and protease inhibitors cocktail (Roche) were disrupted with Lysis Matrix B silica beads in a FastPrep-24 high-speed homogenizer (MP Biomedicals) at 6.0 m/s for 8 cycles with 5 min intervals between each cycle. Protein extracts obtained were purified by phenol precipitation, reconstituted in the lysis/rehydration buffer and, after determining protein concentrations, subjected to 2-DE separation. The first dimension isoelectric focusing (IEF) was conducted using 18 cm IPG strips (pH 4–7) in a Ettan IPGPhor 3 IEF system with the following voltage program: 30 V for 6 h, 60 V for 6 h, 200 V for 1 h, 500 V for 1 h, 1000 V for 1 h, gradient to 8000 V within 30 min and 8000 V for 8 h. Subsequently, the focused IPG strips were equilibrated in two steps of 15 min each with 15 mL of equilibration buffer (50 mM Tris–HCl, pH 8.8, 6 M urea, 30 % w/v glycerol, 2 % w/v SDS) supplemented with 1 % w/v DTT in the first and 2.5 % w/v iodoacetamide in the second step, respectively. The second dimension SDS-PAGE was carried out using 12.5 % polyacrylamide gels with the following running conditions: 1.5 W/gel for 1 h and then 10 W/gel until the bromophenol blue dye front reached the bottom of the gels. After staining with self-made ruthenium II bathophenanthroline disulfonate chelate (RuBPS) fluorescent dye, gels were scanned with a molecular imager (VersaDoc MP4000, Bio-Rad), and gel images were analyzed using the Progenesis SameSpots software v3.3 (Nonlinear dynamic, UK) to detect protein spots showing statistically significant changes in their expression levels before (phase I) and after (phase II) glucose limitation.

Identification of protein spots with significantly changed expression levels was done by nanoLC–ESI–MS/MS analysis using a Ultimate 3000 RSLCnano HPLC system (ThermoFisher Scientific) coupled to an amaZon ETD ion-trap mass spectrometer (Bruker Daltonics). Briefly, after overnight in-gel tryptic digestion at 37 °C, extraction of tryptic peptides and purification with reversed-phased C18 ZipTips (Millipore), tryptic peptides dissolved in 0.1 % TFA were pre-concentrated on a Acclaim PepMap100 C18 (100 µm × 2 cm, 5 µm) column and then separated on a Acclaim PepMap RSLC C18 (75 µm × 15 cm, 2 µm) column. The mobile phases used were A: 0.1 % formic acid in water and B: 10.1 % formic acid in acetonitrile/water (90:10). Peptide were separated using a 30 min linear gradient from 2 to 45 % B delivered at a flow rate of 300 nL/min. Tryptic peptides eluted from the C18 analytical column were introduced into the mass spectrometer through a CaptiveSpray nano-ESI source (Bruker Daltonics) operating at positive mode controlled by using the trapControl acquisition software (version 4.0). The following tuning parameters were used: capillary voltage −1500 V, flow rate and temperature of the drying gas 3 L/min and 160 °C, respectively. The scan range was 300–1500 m/z for MS and 100–2400 m/z for MS/MS. The MS/MS experiments were carried out in data-dependent auto MS/MS mode using a 4 Da window for precursor ion selection and an absolute threshold of 25,000. After the acquisition of 2 MS/MS spectra from the same precursor ion the m/z is excluded from the precursor selection for 1 min. Data acquired from the nanLC–ESI–MS/MS analysis were processed using the Compass DataAnalysis software (version 4.1) to generate XML files, by which only the 300 most intense MS/MS spectra per MS/MS analysis were converted into compounds and used for protein database search. For protein identification the XML files were imported into the ProteinScape software for search against a specific protein database of C. pasteurianum DSMZ 525 installed in-house on a licensed Mascot server. The following parameters were used for protein identification: allow up to 1 missed cleavage, 0.6 Da tolerance both for peptide and MSMS, 1+, 2+ and 3+ peptide charges, carbamidomethyl (C) as fixed moidification, oxidation (M) as variable modification, only accept protein identified by at least 2 peptides with false positive rate <1 %. Samples from two biological replicates [each sample with three technical replicates (3 gels)] were used for proteomic analysis.

### Stoichiometric analysis for energy (ATP), reducing equivalents and product balances

The ATP production as reflected in the product distribution can be characterized as follow:

**Table:** 

Glucose → 2 Acetate + 2 CO_2_ + 4 ATP + 2 NADH_2_ + 2 FdH_2_ Glucose + 2 NADH_2_ → 2 Ethanol + 2 CO_2_ + 2 ATP +2 FdH_2_ Glucose → Butyrate + 2 CO_2_ + 3 ATP + 2 FdH_2_ Glucose + 2 NADH_2_ → Butanol + 2 CO_2_ + 2 ATP + 2 FdH_2_ Butyrate + ATP + 2 NADH_2_ → Butanol	Glycerol → Acetate + CO_2_ + 2 ATP + 2 NADH_2_ + FdH_2_ Glycerol → Ethanol + CO_2_ + ATP + FdH_2_ 2 Glycerol → Butyrate + 2 CO_2_ + 3 ATP + 2 FdH_2_ + 2 NADH_2_ 2 Glycerol → Butanol + 2 CO_2_ + 2 ATP + 2 FdH_2_ Glycerol + NADH_2_ → 1,3-propanediol

$$q_{ATP}^{substrate} \, = \,2q_{acetate } \, + \,q_{ethanol} \, + \,3q_{butyrate} \, + \,2q_{butanol}$$

where $q_{ATP}^{substrate}$ is the specific ATP generation rate from fermentation and is equal to the consumption rate for growth and maintenance in mmol/g*h; $q_{acetate } ,\,q_{ethanol} ,\, q_{butyrate} ,\,q_{butanol}$ are the specific production or consumption rates of the different products in mmol/g*h.

The specific growth rate (µ in h^−1^) and the specific substrate consumption rate (q_s_ in g/g/h) were determined as follows:

$$\mu \, = \,\frac{Ln X2\, - \,LnX1}{t2\, - \,t1}$$

$${\text{q}}_{\text{s}} \, = \,\frac{\mu }{Yxs}$$

where X1 and X2 are the biomass concentrations at time points t1 and t2, and Y_x/s_ is the biomass yield in g_biomass_/g_substrate_.

## Additional file

10.1186/s12934-016-0497-4 Fermentation of mixed substrates by *Clostridium pasteurianum* and its physiological, metabolic and proteomic characterizations.
