# Supplementary material for: Fermentation of mixed substrates by Clostridium pasteurianum and its physiological, metabolic and proteomic characterizations
Source: Microb Cell Fact. 2016 Jun 21;15:114. doi: 10.1186/s12934-016-0497-4 (PMC4916542; doi:10.1186/s12934-016-0497-4)
Supplement: Supplementary file 1 — 10.1186/s12934-016-0497-4 Fermentation of mixed substrates by Clostridium pasteurianum and its physiological, metabolic and proteomic characterizations. [file 12934_2016_497_MOESM1_ESM.docx]

# Supplementary file: Fermentation of mixed substrates by *Clostridium pasteurianum* and its physiological, metabolic and proteomic characterizations

Fig. 1 Supplementary: Products formation in the mixed-substrate fermentations with 10 g/L initial glucose concentration and with gas stripping initiated at 16 h. (BuOH = n-butanol, PDO =1,3-propanediol, HAc = acetate, HBc = butyrate, HFc = Formiate).

Fig. 2 Supplementary: Initial growth rates of *C. pasteurianum* (after 8h incubation) as a function of different carbon sources and different butanol concentrations in anaerobic batch cultures.


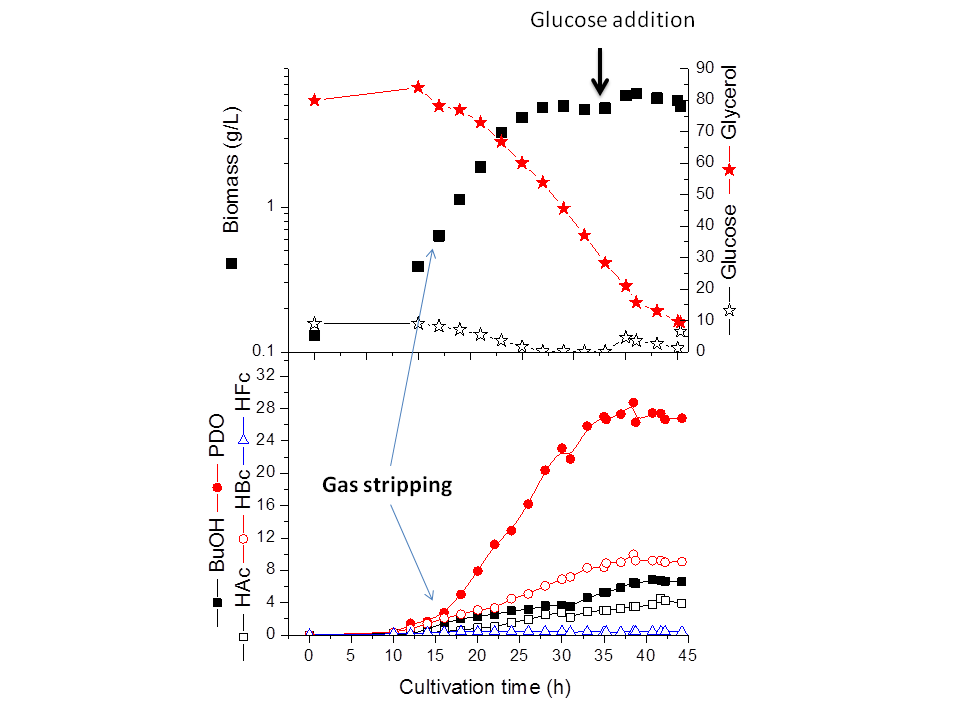


Fig. 1 Supplement

Fig. 2 Supplement

Supplementary Table 1:

Expression level changes of proteins during the course of fermentation with 10g/L initial glucose concentration in the three different phases.

| **Gene name** | **Function or description** | **Normalized value**  **(three gels)** | | | | | | **Fold change II/I** | **Normalized value  (three gels)** | | | **Fold change  III/II** |
| --- | --- | --- | --- | --- | --- | --- | --- | --- | --- | --- | --- | --- |
|  |  | **Phase I** | | | **Phase II** | | |  | **Phase III** | | |  |
| F502_14770 | Serine protein kinase | 6.9E+05 | 7.6E+05 | 5.4E+05 | 8.3E+06 | 5.7E+06 | 1.0E+07 | 12.3 | 1.1E+07 | 6.0E+06 | 1.0E+07 | 1.1 |
|  |  | 2.0E+05 | 1.7E+05 | 1.4E+05 | 5.0E+05 | 2.4E+05 | 6.3E+05 | 2.7 | 4.3E+05 | 3.2E+05 | 6.0E+05 | 1.0 |
|  |  | 6.8E+05 | 6.8E+05 | 6.2E+05 | 4.3E+06 | 2.4E+06 | 4.1E+06 | 5.4 | 4.5E+06 | 3.0E+06 | 4.6E+06 | 1.1 |
| F502_06242 | Chaperonin GroEL | 1.9E+05 | 2.2E+05 | 2.0E+05 | 1.6E+06 | 1.4E+06 | 1.5E+06 | 7.5 | 1.0E+06 | 1.2E+06 | 1.3E+06 | -1.3 |
|  |  | 1.1E+05 | 1.1E+05 | 1.7E+05 | 6.5E+05 | 8.7E+05 | 9.8E+05 | 6.4 | 5.6E+05 | 5.9E+05 | 6.3E+05 | -1.4 |
| F502_00655 | peptidoglycan-binding protein LysM | 4.0E+05 | 5.2E+05 | 2.4E+05 | 1.7E+06 | 2.3E+06 | 3.2E+06 | 6.1 | 1.9E+06 | 2.2E+06 | 1.8E+06 | -1.2 |
| F502_15080 | Rubrerythrin | 1.2E+06 | 7.5E+05 | 9.7E+05 | 5.1E+06 | 5.5E+06 | 5.8E+06 | 5.5 | 8.6E+06 | 9.4E+06 | 8.5E+06 | 1.6 |
| F502_16610 | Glycolate oxidase | 1.3E+05 | 1.8E+05 | 1.8E+05 | 9.5E+05 | 8.6E+05 | 8.9E+05 | 5.5 | 1.3E+06 | 2.0E+06 | 1.8E+06 | 1.9 |
| F502_16565 | nitrogen regulatory protein P-II | 5.8E+05 | 4.8E+05 | 5.2E+05 | 3.2E+06 | 2.2E+06 | 2.9E+06 | 5.3 | 2.9E+06 | 2.6E+06 | 3.5E+06 | 1.1 |
| F502_03342 | Pyruvate phosphate dikinase | 1.5E+05 | 1.2E+05 | 8.9E+04 | 4.9E+05 | 6.1E+05 | 6.4E+05 | 4.9 | 1.4E+06 | 1.2E+06 | 6.9E+05 | 1.9 |
|  |  | 4.7E+04 | 5.0E+04 | 3.4E+04 | 9.8E+04 | 1.5E+05 | 1.5E+05 | 3.0 | 2.7E+05 | 2.8E+05 | 1.4E+05 | 1.8 |
| F502_04232 | Stage IV sporulation protein A | 1.4E+06 | 1.3E+06 | 1.4E+06 | 6.5E+06 | 7.1E+06 | 5.4E+06 | 4.6 | 6.5E+06 | 7.1E+06 | 7.1E+06 | 1.1 |
|  |  | 6.9E+04 | 1.4E+05 | 1.1E+05 | 3.8E+05 | 1.3E+05 | 3.2E+05 | 2.6 | 2.6E+05 | 4.1E+05 | 4.7E+05 | 1.4 |
| F502_18651 | NADP-dependent glyceraldehyde-3-phosphate dehydrogenase | 4.3E+05 | 3.1E+05 | 5.1E+05 | 2.0E+06 | 1.6E+06 | 1.9E+06 | 4.4 | 2.5E+06 | 2.1E+06 | 3.3E+06 | 1.4 |
|  |  | 2.2E+05 | 1.4E+05 | 2.3E+05 | 3.7E+05 | 3.4E+05 | 2.9E+05 | 1.7 | 3.7E+05 | 5.6E+05 | 4.3E+05 | 1.4 |
| F502_06067 | Enolase | 1.1E+05 | 1.3E+05 | 1.1E+05 | 4.4E+05 | 4.8E+05 | 4.2E+05 | 3.8 | 5.0E+05 | 3.5E+05 | 4.1E+05 | -1.1 |
|  |  | 2.6E+06 | 3.5E+06 | 3.2E+06 | 4.9E+06 | 7.1E+06 | 6.2E+06 | 2.0 | 7.3E+06 | 5.1E+06 | 7.4E+06 | 1.1 |
| F502_14780 | SpoVR family protein | 1.4E+05 | 7.8E+04 | 1.3E+05 | 4.1E+05 | 3.5E+05 | 5.3E+05 | 3.7 | 4.8E+05 | 5.4E+05 | 4.9E+05 | 1.2 |
|  |  | 1.4E+05 | 1.1E+05 | 1.6E+05 | 2.3E+05 | 3.0E+05 | 3.0E+05 | 2.0 | 2.3E+05 | 2.9E+05 | 2.5E+05 | -1.1 |
| F502_06247 | Co-chaperonin GroES | 1.3E+06 | 1.5E+06 | 1.6E+06 | 4.6E+06 | 5.3E+06 | 5.2E+06 | 3.5 | 8.9E+06 | 9.1E+06 | 9.7E+06 | 1.8 |
| F502_09238 | Rubredoxin/flavodoxin/oxidoreductase | 6.9E+04 | 5.2E+04 | 6.8E+04 | 2.9E+05 | 1.7E+05 | 1.5E+05 | 3.2 | 2.9E+05 | 3.9E+05 | 3.2E+05 | 1.6 |
|  |  | 3.0E+05 | 2.3E+05 | 3.0E+05 | 1.0E+06 | 7.6E+05 | 7.1E+05 | 3.0 | 1.2E+06 | 1.6E+06 | 1.2E+06 | 1.6 |
| F502_05347 | Putative phosphate starvation-inducible protein PhoH | 6.0E+04 | 5.2E+04 | 4.2E+04 | 1.7E+05 | 1.3E+05 | 1.6E+05 | 3.0 | 1.3E+05 | 1.1E+05 | 1.6E+05 | -1.1 |
| F502_04697 | Phosphocarrier protein (HPr) | 2.4E+05 | 3.4E+05 | 3.1E+05 | 7.8E+05 | 8.2E+05 | 6.9E+05 | 2.6 | 1.1E+06 | 9.8E+05 | 1.3E+06 | 1.5 |
| F502_03987 | Peptidase | 1.3E+05 | 1.0E+05 | 1.1E+05 | 3.2E+05 | 2.6E+05 | 2.3E+05 | 2.4 | 4.9E+05 | 6.0E+05 | 6.1E+05 | 2.1 |
|  |  | 9.2E+04 | 8.2E+04 | 7.1E+04 | 1.5E+05 | 1.3E+05 | 1.2E+05 | 1.6 | 2.5E+05 | 3.5E+05 | 3.0E+05 | 2.2 |
| F502_15100 | Oligoendopeptidase F | 1.9E+05 | 8.1E+04 | 8.2E+04 | 2.9E+05 | 2.7E+05 | 2.8E+05 | 2.4 | 2.7E+05 | 2.8E+05 | 2.7E+05 | 1.0 |
| F502_14060 | Acetoin reductase | 1.2E+05 | 9.4E+04 | 9.6E+04 | 1.9E+05 | 2.6E+05 | 2.6E+05 | 2.3 | 1.7E+05 | 1.5E+05 | 1.5E+05 | -1.6 |
| F502_12878 | Desulfoferrodoxin | 2.5E+05 | 1.7E+05 | 2.7E+05 | 6.3E+05 | 4.0E+05 | 4.6E+05 | 2.2 | 6.4E+05 | 4.5E+05 | 5.2E+05 | 1.1 |
| F502_18446 | Chaperone protein clpb | 4.7E+05 | 1.9E+05 | 3.4E+05 | 7.2E+05 | 7.6E+05 | 6.5E+05 | 2.1 | 1.3E+06 | 7.7E+05 | 7.8E+05 | 1.3 |
| F502_04537 | 30S Ribosomal protein S2 | 9.1E+05 | 1.9E+06 | 9.7E+05 | 2.7E+05 | 3.1E+05 | 2.5E+05 | -4.5 | 3.1E+05 | 3.3E+05 | 1.8E+05 | 1.0 |
| F502_02505 | Biotin synthase | 1.3E+06 | 6.9E+05 | 1.7E+06 | 3.8E+05 | 2.9E+05 | 2.3E+05 | -4.1 | 2.7E+05 | 1.8E+05 | 3.2E+05 | -1.2 |
| F502_11976 | Pyruvate carboxylase | 6.4E+05 | 5.0E+05 | 3.7E+05 | 9.9E+04 | 1.1E+05 | 1.7E+05 | -4.0 | 2.8E+05 | 9.9E+04 | 8.1E+04 | 1.2 |
| F502_07578 | Pyridoxal biosynthesis lyase PdxS | 4.7E+05 | 5.6E+05 | 7.8E+05 | 2.2E+05 | 1.6E+05 | 1.9E+05 | -3.2 | 1.8E+05 | 2.1E+05 | 1.5E+05 | -1.1 |
| F502_04127 | Cell division protein FtsZ | 7.6E+05 | 7.0E+05 | 6.2E+05 | 2.4E+05 | 3.1E+05 | 1.8E+05 | -2.8 | 2.4E+05 | 1.4E+05 | 2.8E+05 | -1.1 |
|  |  | 1.4E+05 | 1.7E+05 | 1.4E+05 | 5.9E+04 | 4.1E+04 | 4.6E+04 | -3.0 | 4.8E+04 | 5.0E+04 | 4.7E+04 | 1.0 |
| F502_08238 | Cell division protein DivIVA | 1.7E+05 | 2.8E+05 | 2.5E+05 | 9.8E+04 | 9.7E+04 | 6.5E+04 | -2.7 | 1.1E+05 | 1.2E+05 | 1.0E+05 | 1.3 |
| F502_07413 | DTPD-D-glucose 4,6 -dehydratase | 1.3E+06 | 1.2E+06 | 1.3E+06 | 5.2E+05 | 5.1E+05 | 4.7E+05 | -2.5 | 3.8E+05 | 3.4E+05 | 5.2E+05 | -1.2 |
| F502_00710 | Gene_pyrG CTP synthetase | 1.9E+05 | 2.1E+05 | 1.1E+05 | 6.5E+04 | 9.9E+04 | 6.2E+04 | -2.3 | 6.0E+04 | 7.6E+04 | 5.4E+04 | -1.2 |
| F502_05017 | NifU related domain containing protein | 5.9E+06 | 4.9E+06 | 7.6E+06 | 3.3E+06 | 2.9E+06 | 2.7E+06 | -2.1 | 2.9E+06 | 2.4E+06 | 2.8E+06 | -1.1 |
| F502_04707 | Adenylosuccinate lyase | 4.9E+05 | 4.8E+05 | 6.0E+05 | 3.1E+05 | 1.7E+05 | 2.8E+05 | -2.0 | 1.9E+05 | 1.2E+05 | 2.5E+05 | -1.4 |
| F502_10588 | Ferritin 50S | 3.7E+05 | 3.9E+05 | 1.9E+05 | 1.8E+05 | 1.2E+05 | 1.6E+05 | -2.0 | 1.2E+05 | 1.3E+05 | 1.3E+05 | -1.2 |
| F502_18843 | Ribosomal protein L7/L12 | 5.5E+06 | 8.1E+06 | 5.1E+06 | 2.5E+06 | 3.7E+06 | 3.2E+06 | -2.0 | 2.5E+06 | 2.5E+06 | 3.1E+06 | -1.2 |
| F502_18848 | DNA-directed RNA polymerase subunit beta | 1.5E+05 | 8.8E+04 | 1.7E+05 | 6.9E+04 | 1.0E+05 | 3.3E+04 | -2.0 | 2.1E+05 | 1.9E+05 | 2.2E+05 | 3.1 |
| F502_18292/18287 | Hydrogene dehydrogenase / hydrogenase -1 | 9.2E+05 | 7.8E+05 | 9.4E+05 | 4.3E+05 | 2.0E+05 | 4.1E+05 | -2.5 | 2.3E+05 | 1.3E+05 | 2.8E+05 | -1.6 |
|  |  | 2.3E+06 | 2.4E+06 | 2.3E+06 | 7.8E+05 | 5.7E+05 | 1.0E+06 | -3.0 | 3.7E+05 | 2.2E+05 | 7.8E+05 | -1.7 |
| F502_03482 | Dihydroxy-acid dehydratase | 6.4E+05 | 7.1E+05 | 4.9E+05 | 3.1E+05 | 2.8E+05 | 3.9E+05 | -1.9 | 2.6E+05 | 2.2E+05 | 2.2E+05 | -1.4 |
| F502_19118 | phosphoenolpyruvate-protein phosphotransferase | 2.6E+05 | 3.9E+05 | 3.2E+05 | 1.4E+05 | 1.7E+05 | 2.3E+05 | -1.8 | 1.5E+05 | 1.6E+05 | 1.6E+05 | -1.2 |

Supplementary Table 2:

Expression level changes of proteins during the course of fermentation with 5 g/L initial glucose concentration in the phases I and II.

| **Gene name** | **Function or description** | **Normalized value** | | | | | | **Fold change** |
| --- | --- | --- | --- | --- | --- | --- | --- | --- |
|  |  | **(three gels)** | | | | | | **II/I** |
|  |  | **Phase I** | | | **Phase II** | | |  |
| F502_14770 | Serine protein kinase | 2.2E+06 | 6.1E+05 | 1.6E+06 | 1.7E+07 | 2.0E+07 | 2.1E+07 | 12.8 |
|  |  | 5.5E+05 | 3.7E+05 | 3.9E+05 | 5.2E+06 | 9.3E+06 | 9.0E+06 | 18.0 |
|  |  | 4.2E+05 | 1.9E+05 | 6.1E+05 | 1.3E+06 | 2.2E+06 | 2.3E+06 | 4.7 |
| F502_00655 | peptidoglycan-binding protein LysM | 2.1E+05 | 5.4E+05 | 4.6E+05 | 5.1E+06 | 5.6E+06 | 4.7E+06 | 12.6 |
| F502_18092 | Stage V sporulation protein T | 8.8E+04 | 9.2E+04 | 1.6E+05 | 1.7E+06 | 1.3E+06 | 1.0E+06 | 12.2 |
| F502_15080 | rubrerythrin | 8.0E+06 | 7.9E+06 | 1.0E+07 | 2.2E+07 | 3.3E+07 | 2.1E+07 | 2.9 |
| F502_07198 | single-stranded DNA-binding protein | 4.1E+05 | 4.9E+05 | 3.8E+05 | 2.1E+06 | 3.4E+06 | 4.2E+06 | 7.5 |
| F502_16610 | Glycolate oxidase | 5.0E+05 | 4.8E+05 | 5.6E+05 | 1.3E+06 | 1.5E+06 | 1.5E+06 | 2.7 |
| F502_16565 | nitrogen regulatory protein P-II | 9.6E+05 | 1.1E+06 | 1.8E+06 | 6.7E+06 | 1.3E+07 | 4.9E+06 | 6.2 |
| F502_03342 | Pyruvate phosphate dikinase | 4.3E+05 | 3.2E+05 | 2.5E+05 | 1.1E+06 | 1.8E+06 | 1.5E+06 | 4.4 |
|  |  | 1.2E+05 | 1.2E+05 | 6.2E+04 | 4.1E+05 | 3.4E+05 | 5.6E+05 | 4.2 |
| F502_04232 | stage IV sporulation protein A | 4.9E+06 | 4.2E+06 | 6.2E+06 | 1.2E+07 | 2.3E+07 | 1.4E+07 | 3.3 |
|  |  | 7.2E+05 | 3.7E+05 | 5.2E+05 | 2.0E+06 | 2.6E+06 | 2.0E+06 | 4.1 |
| F502_18651 | NADP-dependent glyceraldehyde-3-phosphate dehydrogenase | 1.6E+06 | 1.3E+06 | 1.0E+06 | 4.1E+06 | 7.7E+06 | 5.1E+06 | 4.3 |
|  |  | 6.0E+05 | 4.3E+05 | 5.0E+05 | 1.0E+06 | 1.7E+06 | 1.2E+06 | 2.6 |
| F502_14915 | alpha-glucosidase | 3.5E+05 | 3.7E+05 | 3.4E+05 | 8.7E+05 | 1.2E+06 | 1.3E+06 | 3.2 |
| F502_03937 | gene_glgA glycogen synthase | 5.5E+05 | 3.5E+05 | 5.0E+05 | 1.2E+06 | 1.4E+06 | 1.7E+06 | 3.0 |
| F502_06067 | Enolase | 5.1E+06 | 5.5E+06 | 6.9E+06 | 1.1E+07 | 1.7E+07 | 8.7E+06 | 2.1 |
| F502_14780 | SpoVR family protein | 2.0E+06 | 1.6E+06 | 7.6E+05 | 3.6E+06 | 5.0E+06 | 5.2E+06 | 3.2 |
|  |  | 1.0E+06 | 9.6E+05 | 6.6E+05 | 1.7E+06 | 1.6E+06 | 2.0E+06 | 2.0 |
| F502_09238 | Rubredoxin/flavodoxin/oxidoreductase | 5.7E+05 | 6.0E+05 | 5.6E+05 | 1.3E+06 | 1.9E+06 | 2.2E+06 | 3.1 |
|  |  | 9.0E+04 | 9.6E+04 | 1.1E+05 | 2.8E+05 | 3.0E+05 | 4.4E+05 | 3.4 |
| F502_05347 | Putative phosphate starvation-inducible protein PhoH | 2.5E+05 | 2.6E+05 | 1.9E+05 | 9.2E+05 | 1.0E+06 | 9.7E+05 | 4.2 |
| F502_04697 | Phosphocarrier protein (HPr) | 3.6E+05 | 6.6E+05 | 1.0E+06 | 2.2E+06 | 3.1E+06 | 1.2E+06 | 3.2 |
| F502_03987 | Peptidase | 2.7E+05 | 1.7E+05 | 1.5E+05 | 8.1E+05 | 9.4E+05 | 1.1E+06 | 4.8 |
|  |  | 9.1E+04 | 6.5E+04 | 5.7E+04 | 4.9E+05 | 5.4E+05 | 5.4E+05 | 7.4 |
|  |  | 2.9E+04 | 2.7E+04 | 3.5E+04 | 1.1E+05 | 9.3E+04 | 1.6E+05 | 4.0 |
| F502_06447 | bifunctional acetaldehyde-CoA/alcohol dehydrogenase | 5.6E+05 | 5.2E+05 | 3.9E+05 | 7.5E+05 | 1.2E+06 | 1.3E+06 | 2.2 |
| F502_09058 | thiamine pyrophosphate protein central region | 1.0E+05 | 1.3E+05 | 8.4E+04 | 2.9E+05 | 1.9E+05 | 3.3E+05 | 2.6 |
| F502_00410 | Isoleucyl-tRNA ligase | 1.2E+05 | 6.3E+04 | 5.0E+04 | 2.2E+05 | 1.4E+05 | 1.7E+05 | 2.3 |
| F502_05157 | dTDP-4-dehydrorhamnose reductase | 8.1E+05 | 6.9E+05 | 4.6E+05 | 1.7E+06 | 1.5E+06 | 2.2E+06 | 2.8 |
| F502_19151 | Hypothetical protein | 1.1E+05 | 1.2E+05 | 1.4E+05 | 4.0E+05 | 2.7E+05 | 3.0E+05 | 2.6 |
| F502_02505 | Biotin synthase | 4.3E+06 | 4.4E+06 | 6.0E+06 | 2.2E+06 | 2.6E+06 | 1.8E+06 | -2.2 |
| F502_12326 | Transcription accessory protein TEX, RNA-binding protein containing S1 domain | 3.6E+05 | 3.2E+05 | 3.4E+05 | 7.5E+04 | 7.3E+04 | 1.4E+05 | -3.6 |
| F502_07798 | Flagellin | 2.2E+07 | 1.9E+07 | 2.5E+07 | 9.8E+06 | 7.7E+06 | 5.6E+06 | -2.9 |
| F502_07498 | formiminotransferase-cyclodeaminase | 6.4E+05 | 1.0E+06 | 1.4E+06 | 1.9E+05 | 2.1E+05 | 1.6E+05 | -5.4 |
|  |  | 3.4E+05 | 5.0E+05 | 5.3E+05 | 2.1E+05 | 1.4E+05 | 1.3E+05 | -2.9 |
| F502_09488 | hydratase | 3.9E+06 | 3.5E+06 | 1.9E+06 | 8.9E+05 | 9.3E+05 | 1.4E+06 | -2.8 |
| F502_18706 | prolyl-tRNA ligase | 4.6E+05 | 4.9E+05 | 4.2E+05 | 1.6E+05 | 1.3E+05 | 2.1E+05 | -2.7 |
| F502_07578 | Pyridoxal biosynthesis lyase PdxS | 3.0E+06 | 3.6E+06 | 4.6E+06 | 1.7E+06 | 1.1E+06 | 1.4E+06 | -2.6 |
| F502_04127 | Cell division protein FtsZ | 7.3E+05 | 7.2E+05 | 8.0E+05 | 3.6E+05 | 2.4E+05 | 2.6E+05 | -2.6 |
| F502_07413 | DTPD-D-glucose 4,6 -dehydratase | 2.7E+06 | 3.4E+06 | 5.5E+06 | 1.9E+06 | 2.1E+06 | 1.4E+06 | -2.2 |
| F502_00710 | Gene_pyrG CTP synthetase | 7.4E+05 | 7.0E+05 | 6.3E+05 | 3.8E+05 | 1.8E+05 | 3.9E+05 | -2.2 |
| F502_10588 | Ferritin 50S | 8.2E+05 | 1.0E+06 | 1.2E+06 | 2.9E+05 | 4.0E+05 | 1.8E+05 | -3.5 |
| F502_06232 | gene_guaA GMP synthase | 3.1E+06 | 2.9E+06 | 1.8E+06 | 1.2E+06 | 1.3E+06 | 1.5E+06 | -2.0 |
| F502_07643 | pyruvate:ferredoxin (flavodoxin) oxidoreductase, homodimeric | 4.5E+06 | 3.9E+06 | 2.2E+06 | 6.3E+05 | 1.2E+06 | 1.7E+06 | -3.0 |
|  |  | 2.3E+07 | 2.6E+07 | 2.4E+07 | 6.5E+06 | 1.3E+07 | 1.2E+07 | -2.3 |
| F502_03482 | Dihydroxy-acid dehydratase | 1.8E+06 | 1.8E+06 | 1.5E+06 | 7.1E+05 | 5.2E+05 | 9.0E+05 | -2.4 |
| F502_15435 | fructokinase | 2.5E+06 | 2.9E+06 | 4.3E+06 | 1.9E+06 | 1.6E+06 | 1.1E+06 | -2.1 |
| F502_19674 | aspartate kinase | 1.2E+06 | 1.1E+06 | 6.3E+05 | 4.7E+05 | 3.2E+05 | 5.3E+05 | -2.2 |
| F502_12231 | hypothetical protein | 9.7E+05 | 1.4E+06 | 1.8E+06 | 7.5E+05 | 5.2E+05 | 5.1E+05 | -2.3 |
| F502_19118 | phosphoenolpyruvate-protein phosphotransferase | 7.0E+05 | 7.8E+05 | 1.1E+06 | 3.7E+05 | 2.5E+05 | 5.2E+05 | -2.3 |
| F502_06272 | ferredoxin-NADP(+) reductase subunit alpha | 1.6E+06 | 1.7E+06 | 2.3E+06 | 8.3E+05 | 6.2E+05 | 4.7E+05 | -2.9 |
| F502_14710 | hypothetical protein | 1.8E+06 | 2.2E+06 | 2.6E+06 | 1.1E+06 | 9.6E+05 | 8.4E+05 | -2.3 |
| F502_12241 | hypothetical protein | 3.7E+06 | 3.5E+06 | 2.9E+06 | 1.3E+06 | 1.4E+06 | 1.8E+06 | -2.3 |
